# Supplementary material for: Iridal-Type Triterpenoids Displaying Human Neutrophil Elastase Inhibition and Anti-Inflammatory Effects from Belamcanda chinensis
Source: Molecules. 2021 Oct 31;26(21):6602. doi: 10.3390/molecules26216602 (PMC8588536; doi:10.3390/molecules26216602)
Supplement: Supplementary file 1 [file molecules-26-06602-s001.zip › Supplementary materials-newest version-1.pdf]

Supplementary material

Iridal-type triterpenoids displaying human neutrophil elastase inhibition and anti-inflammatory effects from *Belamcanda chinensis*

▶Characterization Data

- Figure. S1−7: NMR and HRESIMS data of compound **1**
- Figure. S8−14: NMR and HRESIMS data of compound **2**
- Figure. S15−21: NMR and HRESIMS data of compound **3**
- Table S1: Primer sequences used for RT- PCR

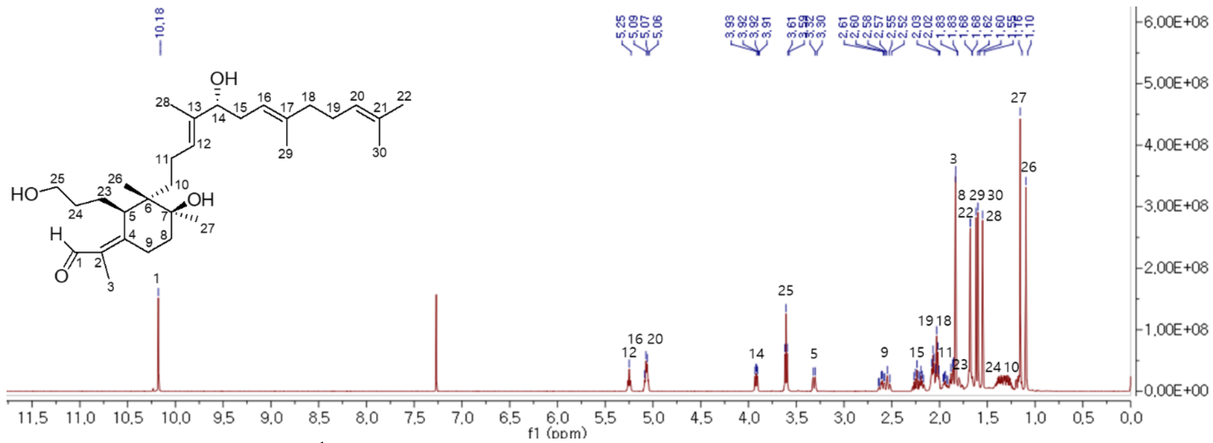

Figure. S1. ^{1}H-NMR spectrum of compound **1** (500 MHz, chloroform-*d*)

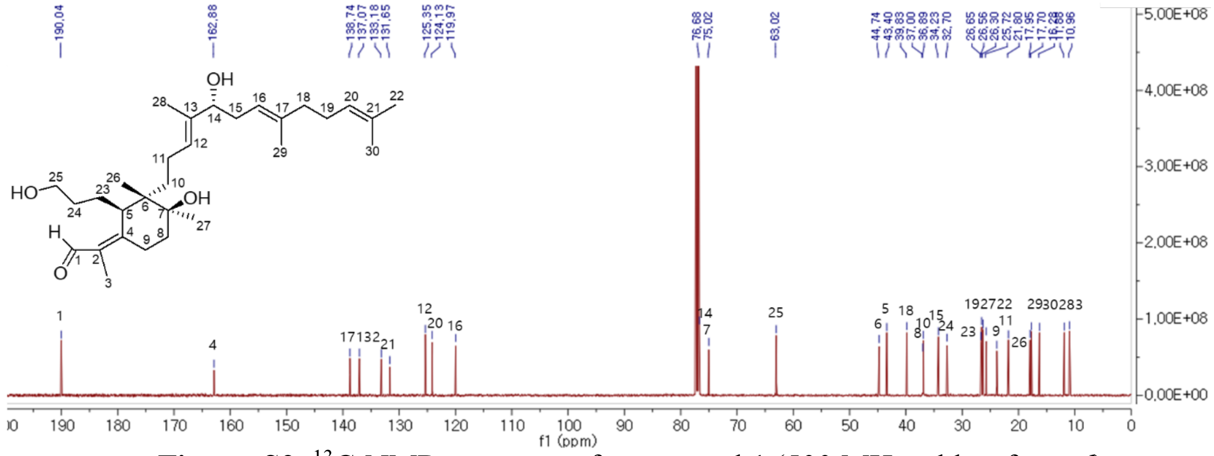

Figure. S2. ^{13}C-NMR spectrum of compound **1** (500 MHz, chloroform-*d*)

DEPT 90

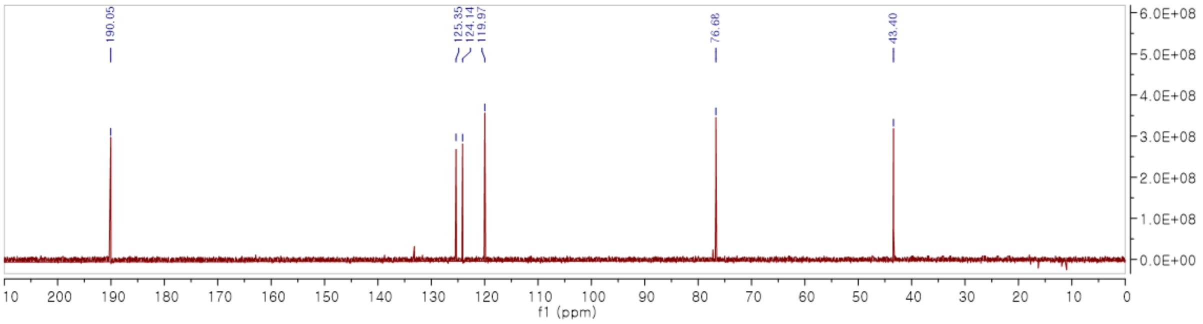

DEPT 135

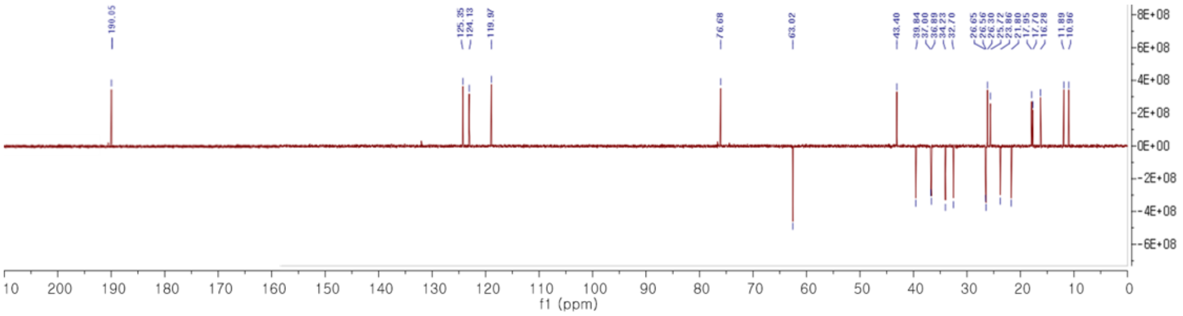

Figure. S3. DEPT-90 and **-**135 spectra of compound **1**.

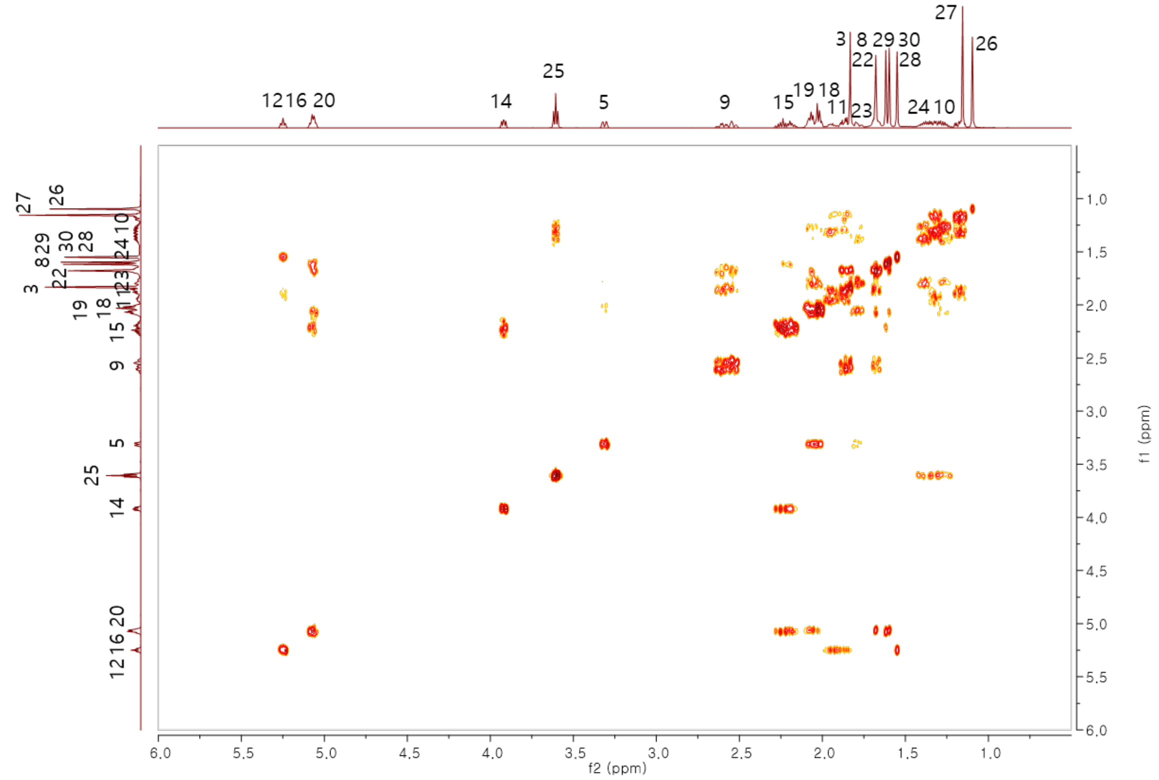

Figure. S4. COSY spectrum of compound **1**.

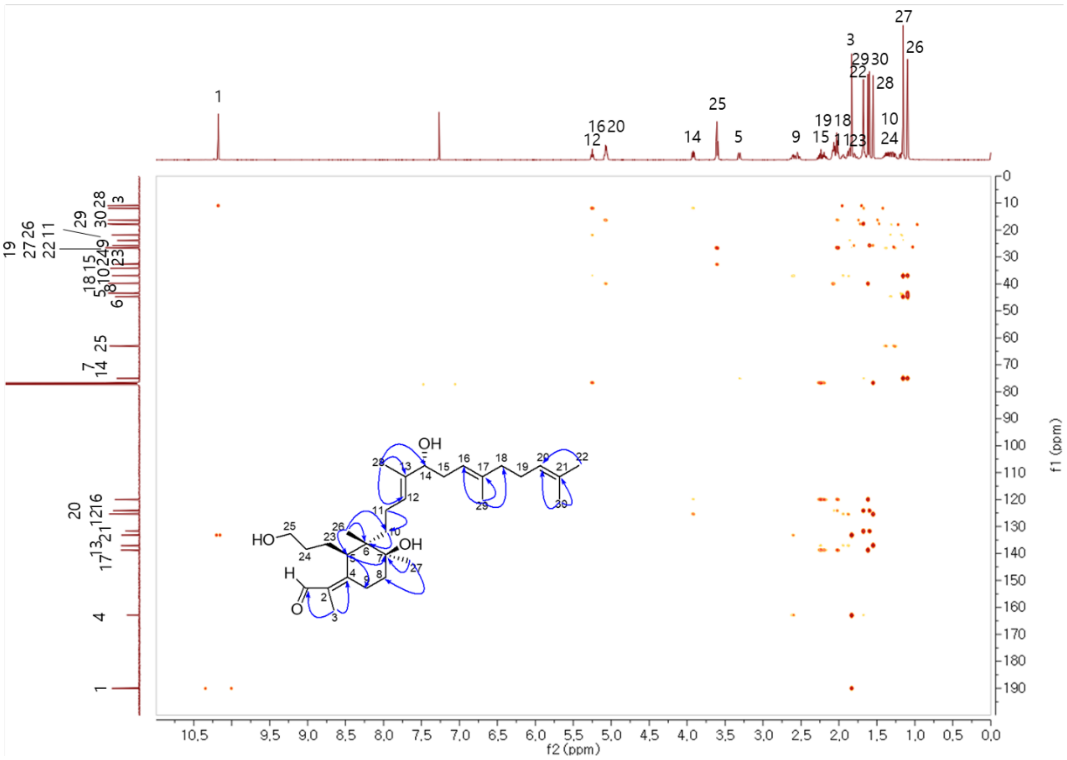

Figure. S5. HMBC spectrum of compound **1**.

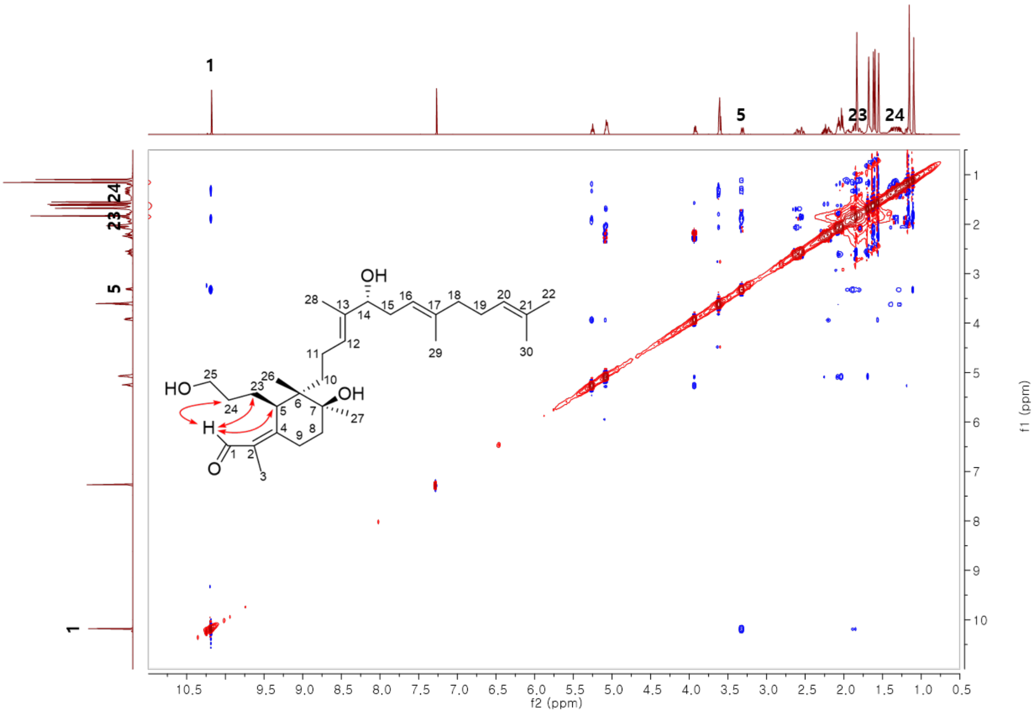

Figure. S6. NOESY spectrum of compound **1**.

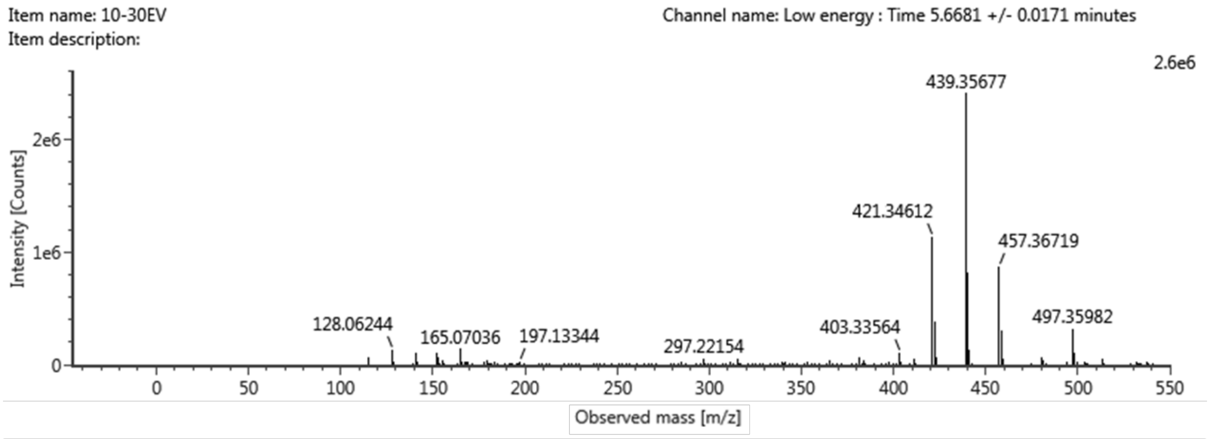

Component name	Formula	Neutral mass (Da)	Observed neutral mass (Da)	Observed m/z	Mass error (mDa)	Mass error (ppm)	Observed RT (min)	Adducts
Compound 1	$C_{30}H_{50}O_4$	474.37091	474.3706	497.3598	-0.3	-0.6	5.67	+Na

Figure. S7. LC-Q-TOF/MS data of compound **1**.

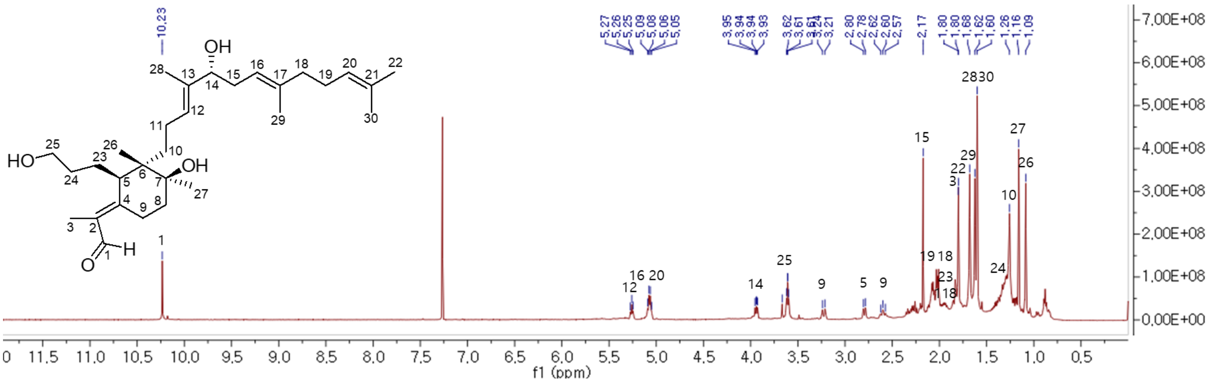

Figure. S8. ^{1}H-NMR spectrum of compound **2** (500 MHz, chloroform-*d*)

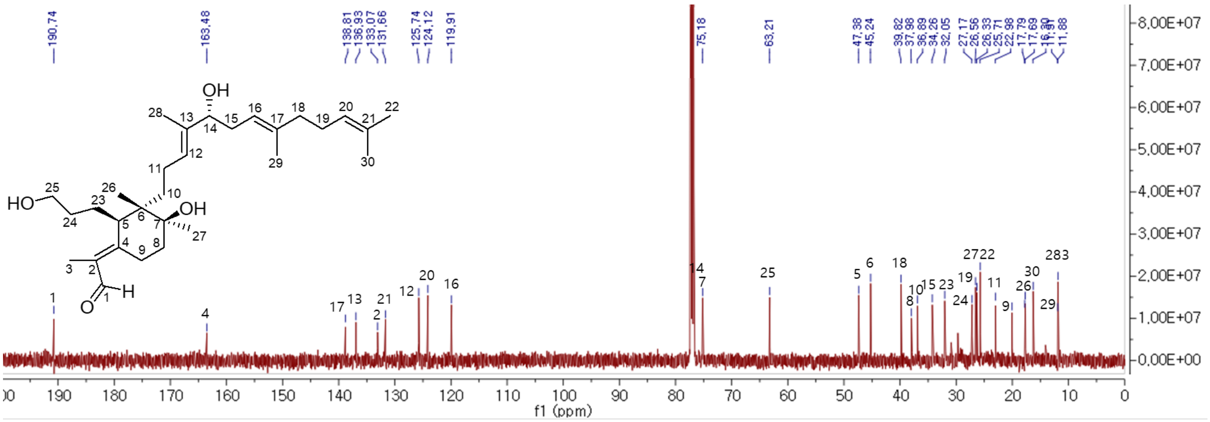

Figure. S9. ^{13}C-NMR spectrum of compound **2** (500 MHz, chloroform-*d*)

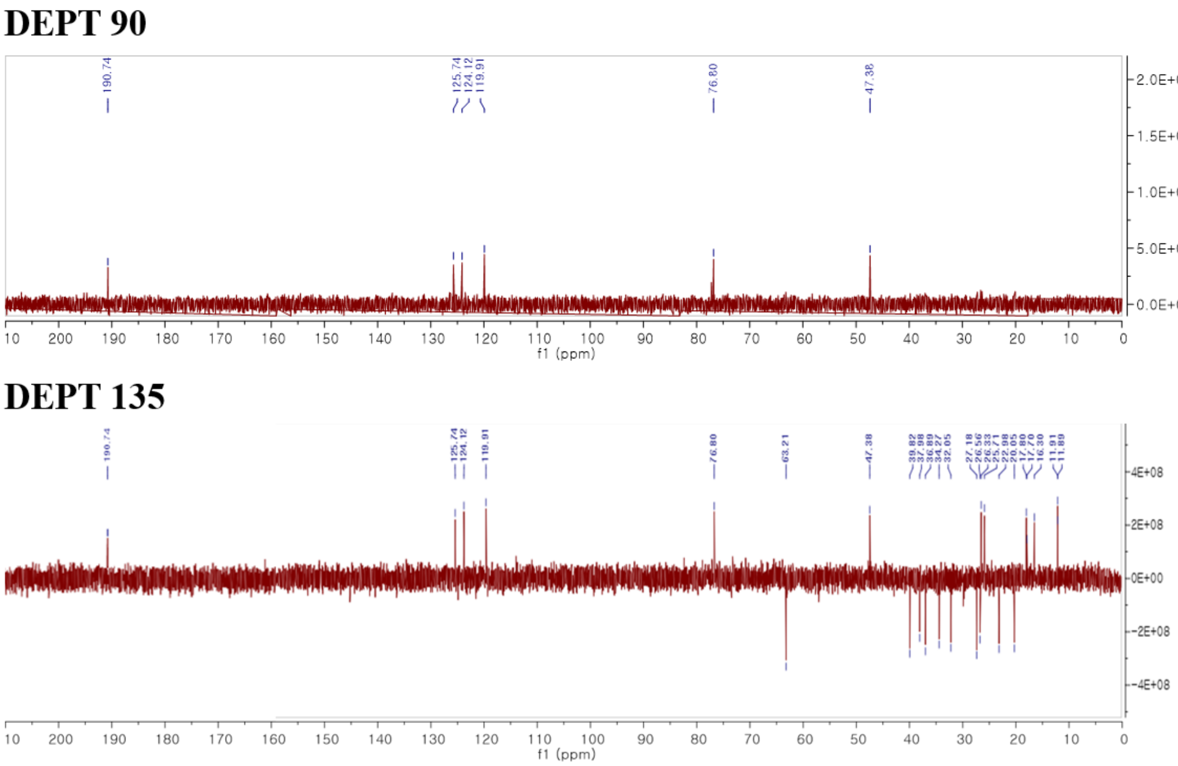

Figure. S10. DEPT-90 and -135 spectra of compound **2**.

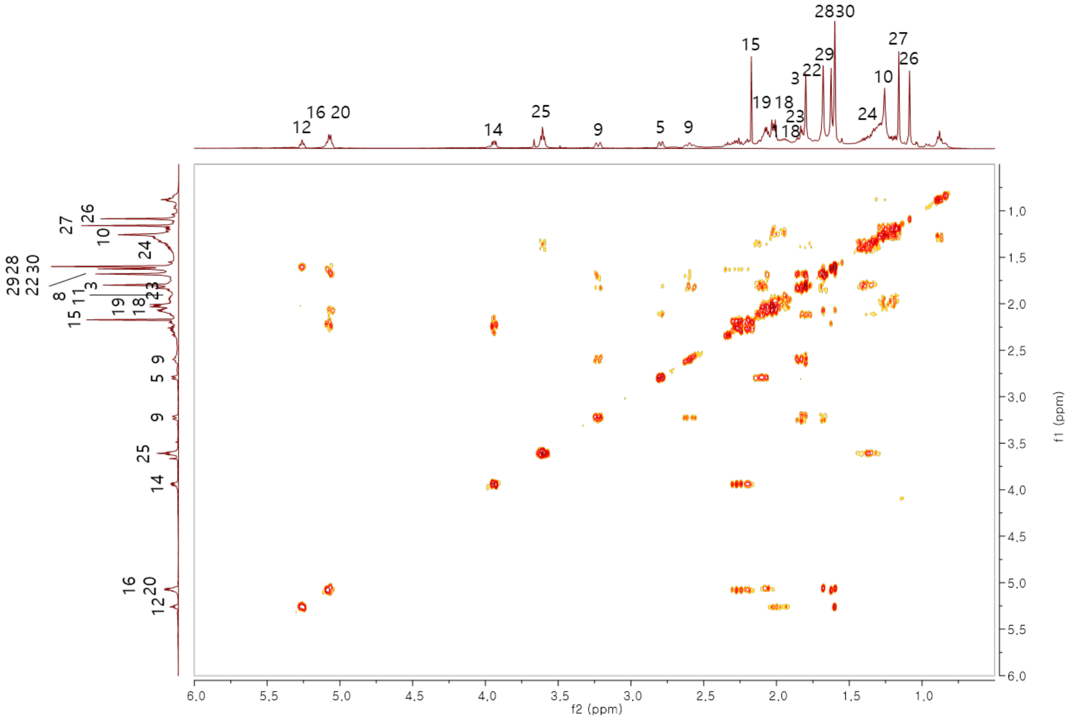

Figure. S11. COSY spectrum of compound **2**.

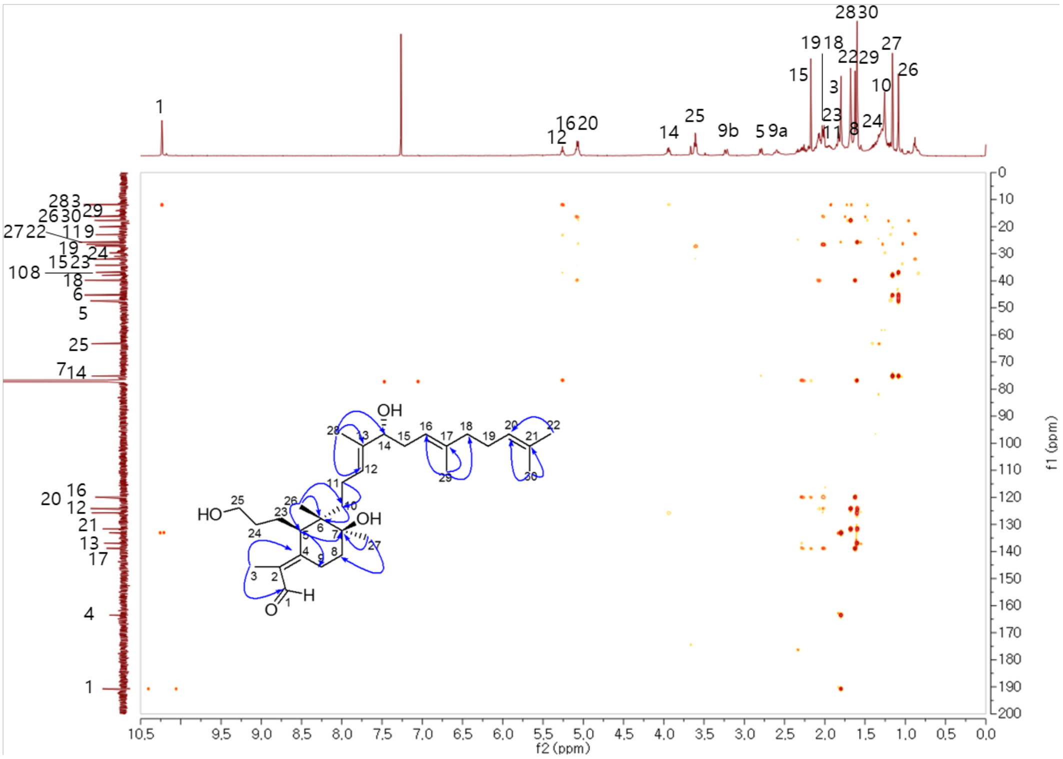

Figure. S12. HMBC spectrum of compound **2**.

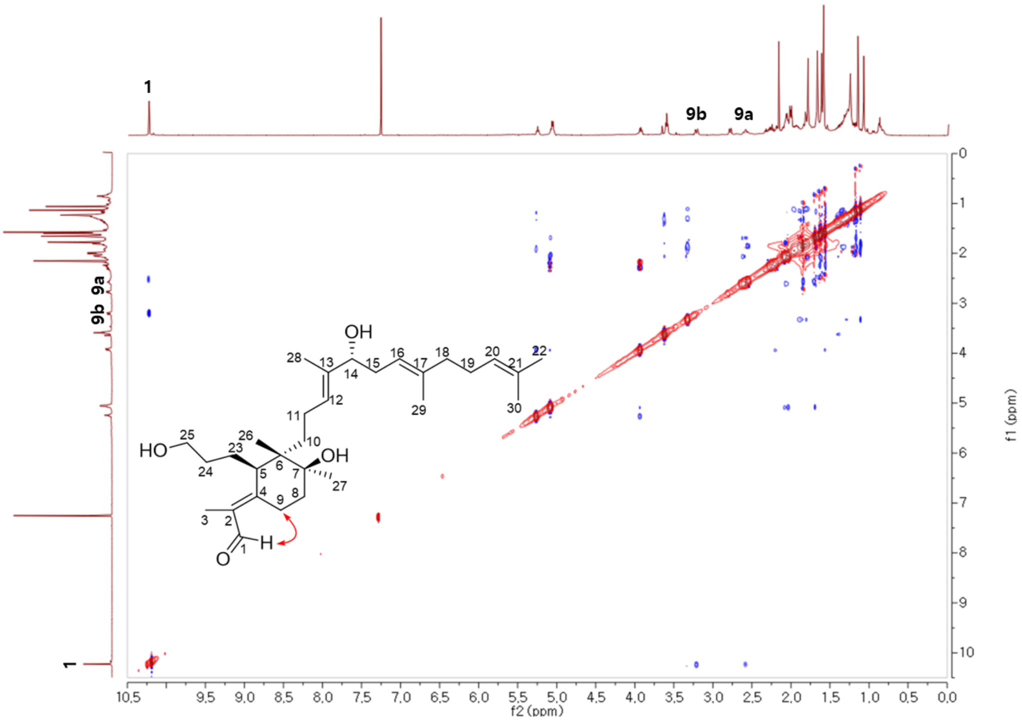

Figure. S13. NOESY spectrum of compound **2**.

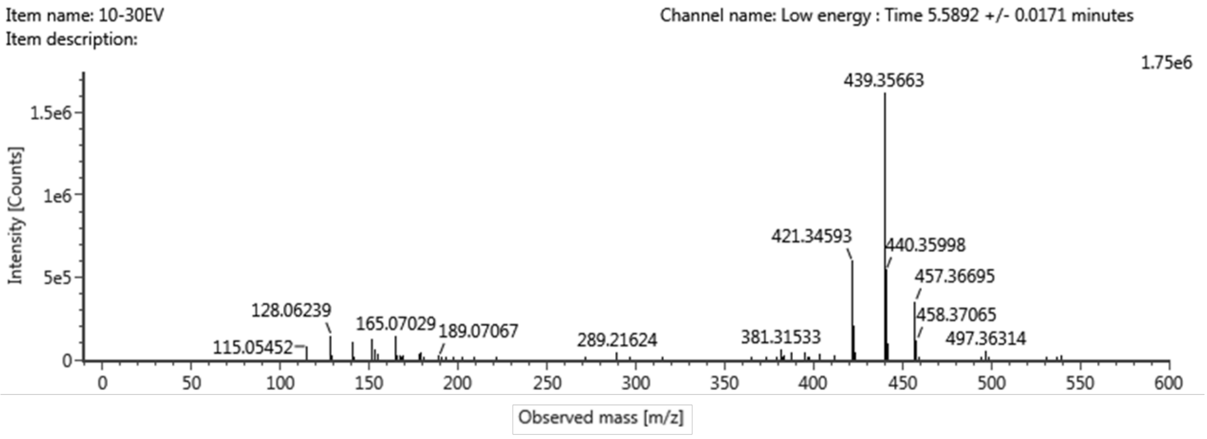

Component name	Formula	Neutral mass (Da)	Observed neutral mass (Da)	Observed m/z	Mass error (mDa)	Mass error (ppm)	Observed RT (min)	Adducts
Compound 2	$C_{30}H_{50}O_4$	474.37091	474.3739	497.3631	3.1	6.3	5.59	+Na

Figure. S14. LC-Q-TOF/MS data of compound **2**.

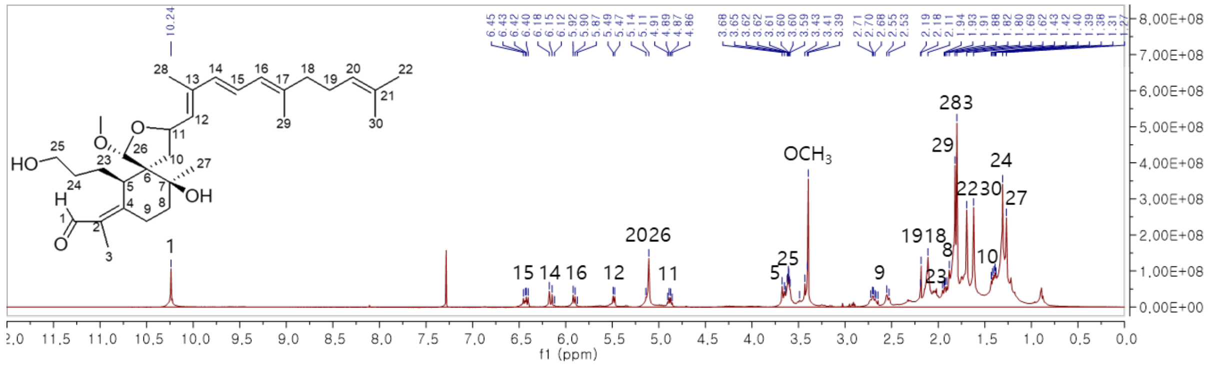

Figure. S15. ^{1}H-NMR spectrum of compound **3** (500 MHz, chloroform-*d*).

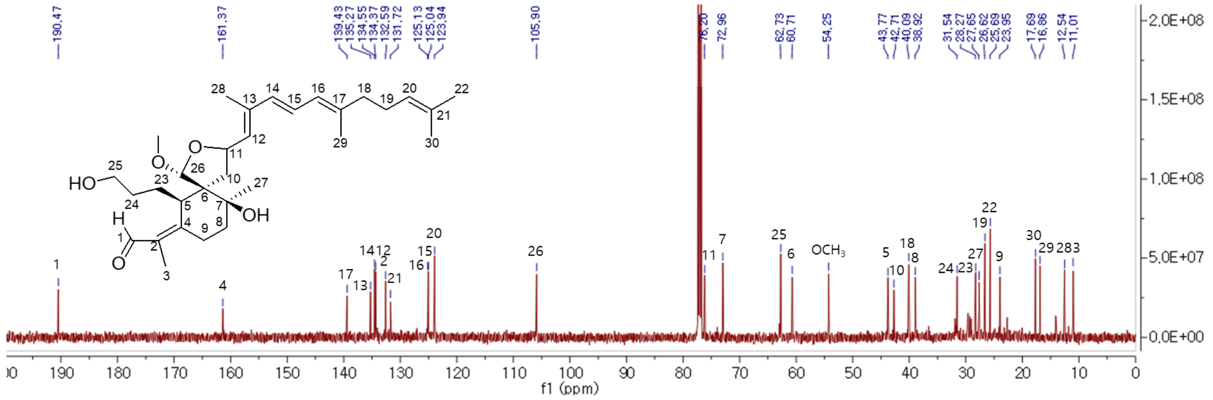

Figure. S16. ^{13}C-NMR spectrum of compound **3** (500 MHz, chloroform-*d*).

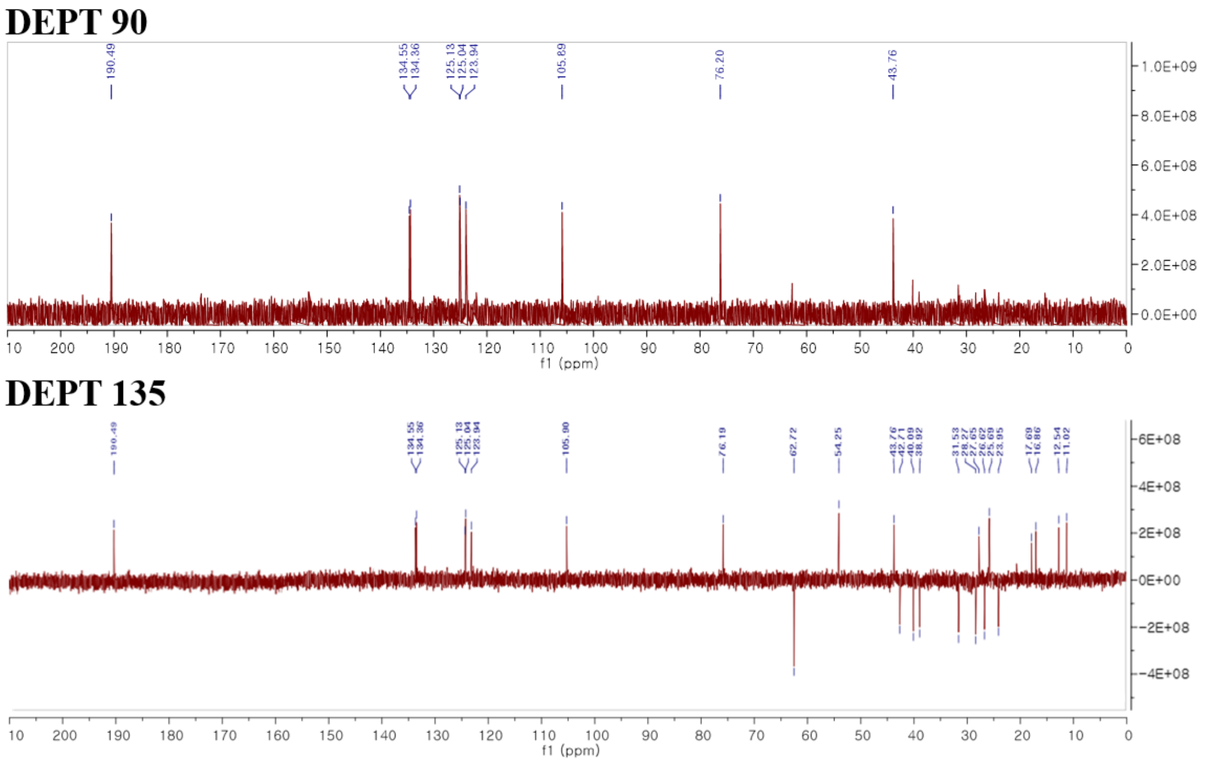

Figure. S17. DEPT-90 and -135 spectra of compound **3**.

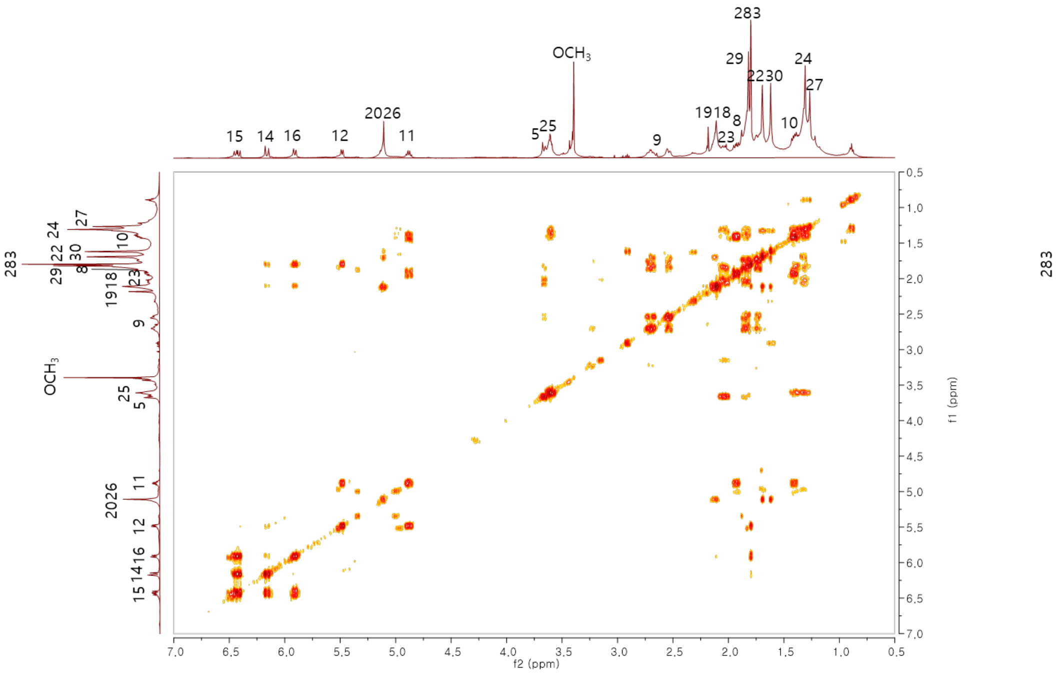

Figure. S18. COSY spectrum of compound **3**.

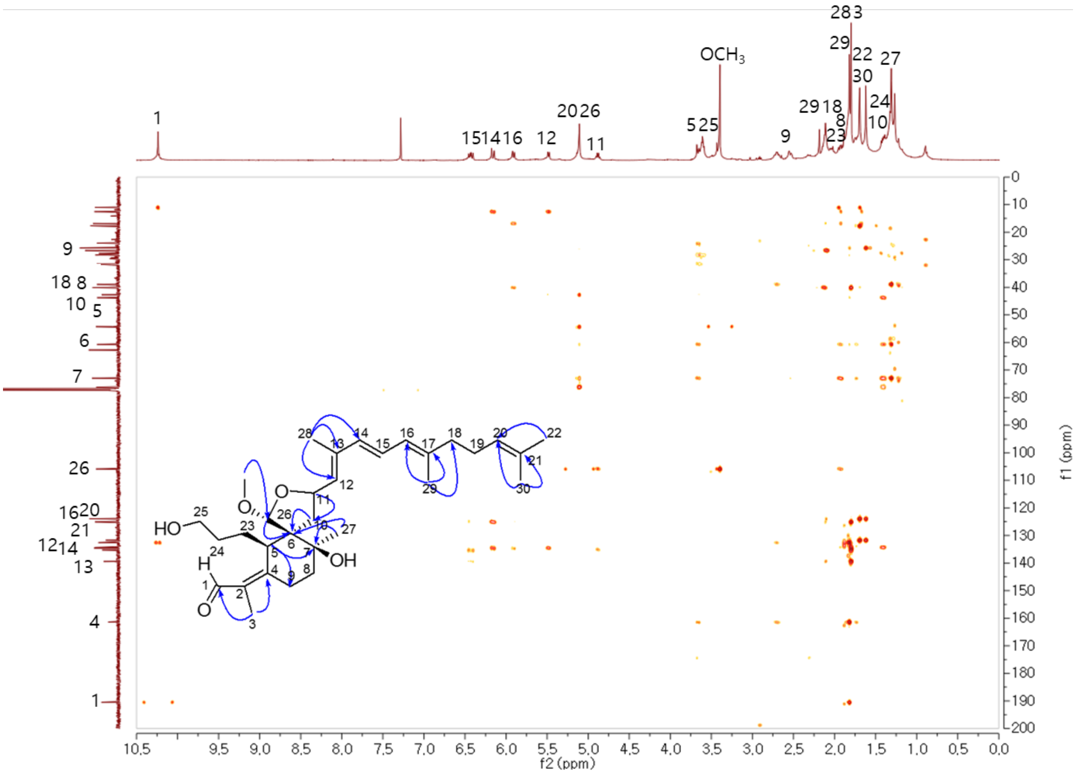

Figure. S19. HMBC spectrum of compound **3**.

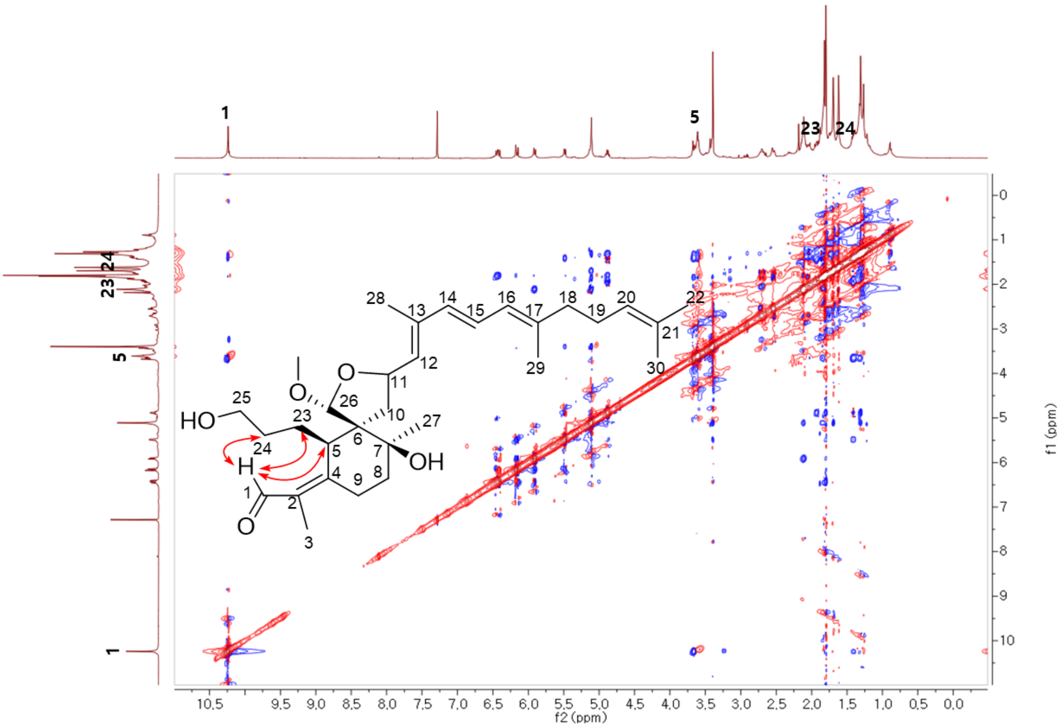

Figure. S20. NOESY spectrum of compound **3**.

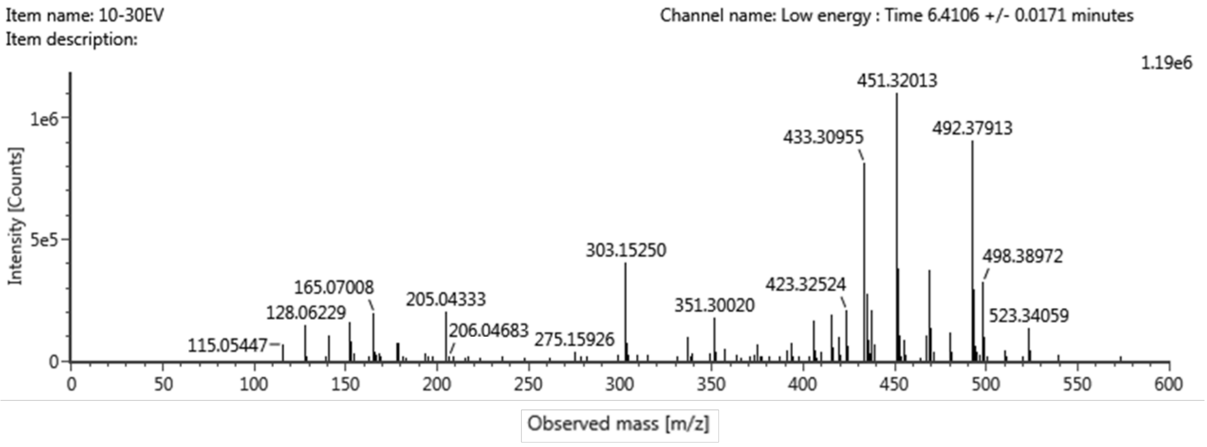

Component name	Formula	Neutral mass (Da)	Observed neutral mass (Da)	Observed m/z	Mass error (mDa)	Mass error (ppm)	Observed RT (min)	Adducts
Compound 4	$C_{31}H_{48}O_5$	500.35017	500.3514	523.3406	-0.3	0.6	6.41	+Na

Figure. S21. LC-Q-TOF/MS data of compound **3**.

Table S1. Primer sequences used for RT- PCR

Gene Name	GenBank Acc. No.	Primer Sequences (5′–3′)	Expected Size (bp)
Il1b	NM_008361.4	Sense: GTTGACGGACCCCAAAAGAT	440
		Antisense: TCGTTGCTTGGTTCTCCTTG	
Nos2	NM_012611.3	Sense: CTCCATGACTCTCAGCACAGAG	401
		Antisense: GCACCGAAGATATCCTCATGAT	
Tnf	NM_013693.3	Sense: CAGCCTCTTCTCTTCTCATTCCTGC	339
		Antisense: TGTCCCTTGAAGAGAGAACCTG	
Gapdh	GU214026	Sense: CTA AAG GGC ATC CTG GGC	201
		Antisense: TTA CTC CTT GGA GGC CAT	
